# A5 A MOUSE MODEL TO UNRAVEL THE PATHOPHYSIOLOGICAL LINK BETWEEN CROHN’S DISEASE AND TYPE-2 DIABETES-ASSOCIATED METABOLIC DISORDERS

**DOI:** 10.1093/jcag/gwac036.005

**Published:** 2023-03-07

**Authors:** T Mukherjee, J Yadav, N Nathan, D Tsang, A Yan, S Cash, C Cummins, P Vlachou, S Girardin, D Philpott

**Affiliations:** 1 Department of Immunology/ Laboratory Medicine and Pathobiology; 2 Department of Immunology; 3 Department of Pharmaceutical Sciences; 4 Department of Medical Imaging, University of Toronto, Toronto, Canada

## Abstract

**Background:**

Crohn’s disease (CD), an idiopathic inflammatory bowel disease (IBD), has been recently shown to increase the risk of developing type 2 diabetes (T2D). Moreover, treatment with anti-diabetic drugs has a protective role in preventing the severity and course of CD progression. However, the pathophysiological basis of T2D development in CD remains unclear. Findings have highlighted the contribution of adipose tissue (AT) to the development of chronic inflammatory diseases and have identified parallels between T2D and CD that may provide hints to common mechanisms of disease pathogenesis. Typically, microbial dysbiosis, hyperpermeable intestinal barrier, and intra-abdominal AT accumulation are the common features of both diseases, yet how the interplay of these factors contribute to pathogenesis is not known. Therefore, common pathogenic paradigms underlying both T2D and CD have led us to hypothesize that chronic intestinal inflammation serves as an initiator of AT dysfunction in CD, predisposing individuals to T2D. Further, the lack of appropriate animal models of CD with chronic intestinal inflammation that manifests accumulation of intra-abdominal AT, and extra-intestinal metabolic disorder as observed in CD and T2D patients has been a limitation.

**Purpose:**

To develop a genetic mouse model to investigate if gut inflammation-mediated microbial dysbiosis and metabolic dysregulation of AT are at the nexus that cause T2D in CD.

**Method:**

We developed a CD-mouse model, where we challenged Nod2-deficient mice (NOD2 being the strongest genetic risk factor contributing to CD) with a chronic inflammatory insult regime, using dextran sulfate sodium (cDSS) for 3 cycles. Subsequently, intraperitoneal insulin and oral glucose tolerance tests, metabolic caging, and MRI imaging of mice were performed. Changes in AT metabolism and microbial infiltration into AT were analyzed by quantitative real-time PCR (qRT-PCR) and/or immunohistochemistry (IHC).

**Result(s):**

Our new CD-mouse model revealed increased gut inflammation (TNF and type-I IFN) in Nod2-deficient mice compared to wild-type control mice post-cDSS. Surprisingly, Nod2-deficient mice gained body weight, which was at least in part accounted for by an increased intra-abdominal AT accumulation along with decreased AT fatty-acid metabolism (*Cpt1a*, *Fabp4* expression) and AT browning (*Ucp1*, *Cidea* expression, and UCP-1 staining), reduced intestinal goblet cell numbers, increased gut bacterial infiltration within the fat, more insulin resistance and energy expenditure.

**Conclusion(s):**

This experimental mouse model mimicking CD-associated T2D will provide insights into how the microbiome-AT axis fuel chronic inflammation-mediated extra-intestinal metabolic disorder and immune dysregulation. Understanding these connections will be transformative, as it will help us devise novel therapeutic strategies to prevent T2D development in progressive CD patients.

**Disclosure of Interest:**

None Declared

